# Subclinical thyroid dysfunction and risk of carotid atherosclerosis

**DOI:** 10.1371/journal.pone.0182090

**Published:** 2017-07-27

**Authors:** Hosu Kim, Tae Hyuk Kim, Hye In Kim, So Young Park, Young Nam Kim, Seonwoo Kim, Min-Ji Kim, Sang-Man Jin, Kyu Yeon Hur, Jae Hyeon Kim, Moon-Kyu Lee, Yong-Ki Min, Jae Hoon Chung, Mira Kang, Sun Wook Kim

**Affiliations:** 1 Division of Endocrinology & Metabolism, Department of Medicine, Thyroid Center, Samsung Medical Center, Sungkyunkwan University School of Medicine, Seoul, Korea; 2 Division of Endocrinology, Department of Medicine, Gyeongsang National University Changwon Hospital, Changwon, Korea; 3 Statistics and Data Center, Samsung Biomedical Research Institute, Seoul, Korea; 4 Center for Health Promotion, Samsung Medical Center, Seoul, Korea; The University of Tokyo, JAPAN

## Abstract

**Background:**

The effect of subclinical thyroid dysfunction on vascular atherosclerosis remains uncertain. The objective of this study was to elucidate the association between sustained subclinical thyroid dysfunction and carotid plaques, which are an early surrogate marker of systemic atherosclerosis.

**Methods:**

The study included 21,342 adults with consistent thyroid hormonal status on serial thyroid function tests (TFTs) and carotid artery duplex ultrasonography at a health screening center between 2007 and 2014. The effect of subclinical thyroid dysfunction on baseline carotid plaques and newly developed carotid plaques during 5-year follow-up was determined by logistic regression analyses and GEE (Generalized Estimating Equations), respectively.

**Results:**

Carotid plaques were more common in the subclinical hypothyroidism (55.6%) than the euthyroidism (47.8%) at baseline. However, in multivariable analysis, thyroid status was not a significant risk for the carotid plaques at baseline. Instead, traditional cardiovascular risk factors, such as age (*P* <0.001), systolic blood pressure (*P* = 0.023), fasting blood glucose (*P* = 0.030), and creatinine (*P* = 0.012) were associated with baseline carotid plaques in subclinical hypothyroidism. In longitudinal analyses of subjects who were followed up for more than 5 years, there was no significant difference in the cumulative incidence of new carotid plaques according to time between subjects with subclinical hypothyroidism and those with euthyroidism (*P* = 0.392).

**Conclusions:**

Sustained subclinical thyroid dysfunction did not affect the baseline or development of carotid plaques in healthy individuals.

## Introduction

The influence of overt thyroid dysfunction on the cardiovascular system is well documented [1–3]. Many studies have also been conducted in subjects with subclinical thyroid dysfunction; however, the effect of subtle thyroid dysfunction on vascular atherosclerosis remains controversial [4–7]. Because of the high prevalence of subclinical hypothyroidism and subclinical hyperthyroidism (4–10% and 0.7–12% of the general population respectively) [8–10], it is important to clarify the effect of subclinical thyroid dysfunction on vascular atherosclerosis.

Carotid plaques are an early surrogate marker of systemic atherosclerosis and a predictor of major cardiovascular events [11]. Many previous studies have tried to evaluate the association between subclinical thyroid dysfunction and incidence of carotid plaques [6,12]. However, most studies utilized a single set of thyroid function tests for analysis of the association between thyroid function and carotid atherosclerosis. In addition, only a few studies reported longitudinal assessment of the change in carotid atherosclerosis burden. In subclinical hyperthyroidism, 40–60% of cases return to normal thyroid function over a period of weeks to a year [13,14]. Moreover, only 33–55% of subclinical hypothyroidism cases progress to overt hypothyroidism during 10 to 20 years of follow-up [15–17]. Therefore, using a single set of baseline thyroid function tests (TFTs) may result in inclusion of many cases of transient subclinical thyroid dysfunction. And it may dilute the probable effect of subclinical thyroid dysfunction on carotid atherosclerosis. Also, in order to evaluate the long-term effects of subclinical thyroid dysfunction on carotid atherosclerosis, serial measurement of TFT is needed.

Here, we aimed to clarify the association between sustained subclinical thyroid dysfunction and carotid atherosclerosis through cross sectional and longitudinal assessment using data from comprehensive health checkups of adults.

## Methods

### Study population

The authors retrospectively screened a total of 23,724 subjects aged 20 or older who underwent more than two comprehensive health checkup examinations including serum TFT and carotid artery duplex ultrasonography (DUS) at Samsung Medical Center between January 2007 and December 2014. All subjects visited the hospital voluntarily for a health checkup. This study was approved by the Institutional Review Board of Samsung Medical Center (IRB File No. 2016-05-112). IRB waived the consent form because all data was anonymized and no personal information was included.

**Fig 1:**
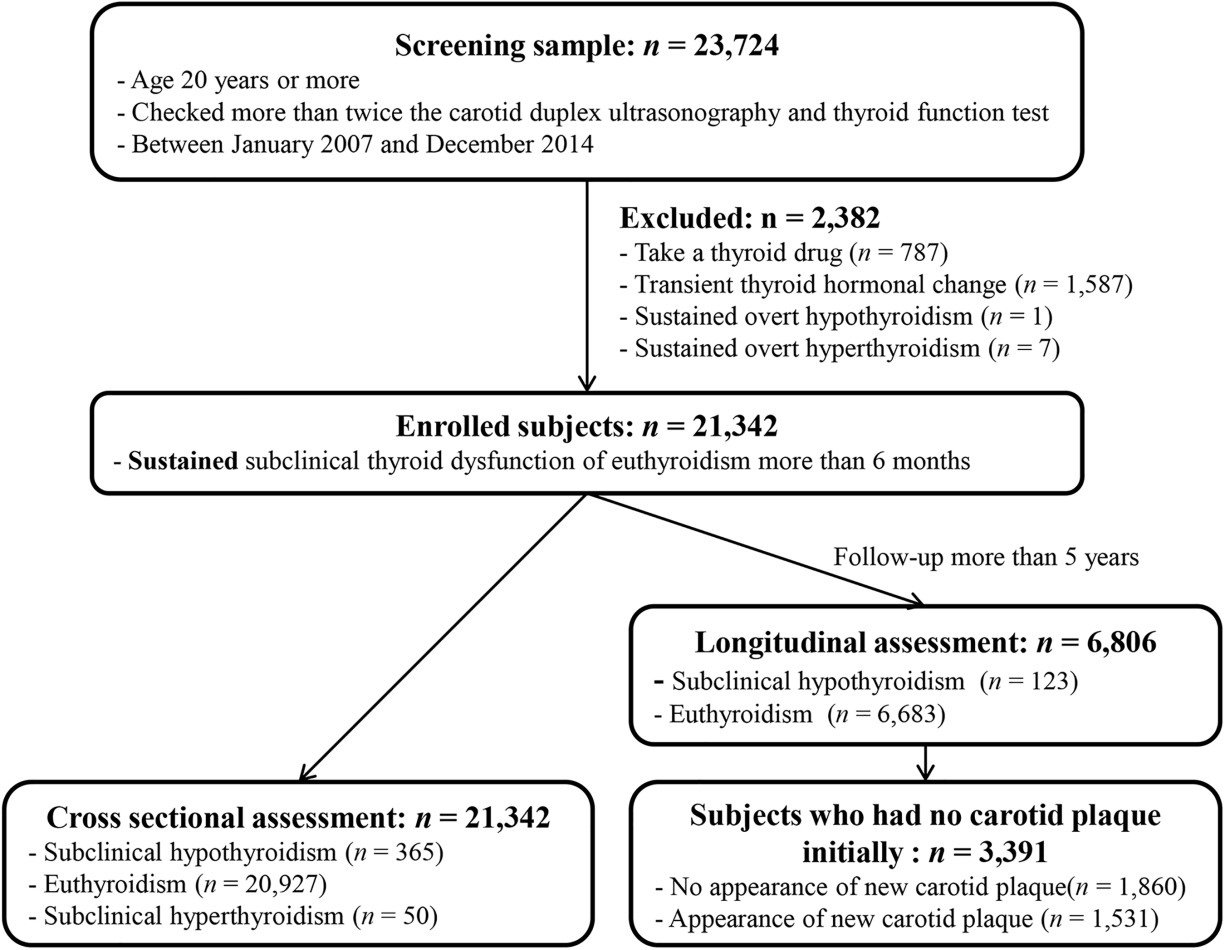
Flow diagram of study subjects. Cross-sectional assessment of carotid plaques was performed for the 21,342 enrolled subjects; of these, 3,391 subjects who showed sustained thyroid functional status with at least 5 years of follow-up were screened for longitudinal assessment of new carotid plaques.

Among subjects, 4.3% and 1.3% showed subclinical hypothyroidism and subclinical hyperthyroidism at initial TFT, and 92.4% showed euthyroid status. We defined sustained thyroid functional status as consistent serial TFT results at a ≥6-month interval. By this definition, subjects with transient thyroid dysfunction were excluded (n = 1,587). Subjects who had been treated for thyroid disease initially were also excluded (n = 787). If a subject started medication for thyroid disease during follow-up, data were included only up to that point. One subject with overt hypothyroidism and seven subjects with overt hyperthyroidism were also excluded (Fig 1). Cross-sectional assessment of carotid plaques at baseline was performed for the remaining 21,342 subjects: 365 had subclinical hypothyroidism, 20,927 had euthyroidism, and 50 had subclinical hyperthyroidism.

For longitudinal assessment of new carotid plaques, 3,391 subjects with no initial carotid plaques and at least 5 years of follow-up were screened. Among them, 1,860 subjects remained free of plaques and 1,531 subjects developed new carotid plaques during follow-up (Fig 1). The subjects with subclinical hyperthyroidism were excluded due to small number of cases (n = 50).

### Measurement of clinical and laboratory variables

Medical history and social behavioral information were collected through questionnaires completed by the subjects. Exercise status was defined as regular exercise of at least moderate intensity more than three times per week. Drinking status was defined as consuming more than 20 g of alcohol per week. Height and weight were measured for the calculation of body mass index [BMI = weight (kg)/height (m)^2^]. Systolic blood pressure (SBP) and diastolic blood pressure (DBP) were measured in seated subjects after a 15-min rest period. Metabolic syndrome was defined according to the US National Cholesterol Education Program/Adult Treatment Panel III guideline (NCEP/ATP III) [18].

Serum-free T4 (FT4), total T3 (TT3), and thyroid stimulating hormone (TSH) were measured by chemiluminescent immunoassay (ADVIA Centaur® XP; Seimens). An enzymatic colorimetric method was used to measure serum total cholesterol (TC) and triglyceride (TG). Low-density lipoprotein cholesterol (LDL-C) and serum high-density cholesterol were measured by a homogenous enzymatic colorimetric method (Roche/Hitachi modular; Roche). Fasting blood sugar (FBS), hemoglobin A1c (HbA1c), serum creatinine, C-reactive protein (CRP), and liver function tests such as alanine transaminase (ALT) and asparatate aminotransferase (AST) were also checked.

Carotid DUS was used to measure carotid atherosclerosis. For the analysis, the authors checked for abnormal carotid plaques using a 4.4-MHz pulsed Doppler (LOGIQ E9; GE). Carotid plaque was defined as focal carotid intima-media thickness >1.5 mm or a vessel wall that appeared to be at least 50% thicker than the surrounding wall. We performed the measurements at carotid bifurcation on the far wall of the common carotid artery on both sides and the computer-based points in the region was used in this study [19,20].

### Definition of thyroid functional status

Thyroid hormone status was defined as serum TSH and FT4 levels sustained for more than 6 months. Normal TSH level ranged from 0.3 to 6.0 mU/L and normal FT4 level ranged from 0.8 to 1.85 ng/dL that were institutional reference ranges of our center [21]. Euthyroidism was defined as TSH and FT4 levels in the normal range. Subclinical hypothyroidism was defined as TSH level greater than 6 mU/L, and subclinical hyperthyroidism as TSH level less than 0.3 mU/L. For the subgroup analysis, severe subclinical hypothyroidism was defined as TSH >10 mU/L and normal FT4 level.

### Statistical analysis

Continuous data were expressed as the mean ± standard deviation (SD) as indicated. Data on categorical characteristics were expressed as percent values or absolute numbers as indicated. Continuous data with no normality were expressed as the median and inter-quartile range. For comparison among the three groups of thyroid functional status, χ^2^-test was used for categorical data and ANOVA or Kruskal-Wallis test was used for continuous data. Tukey’s test was used for post-hoc analysis. For univariable and multivariable analysis, logistic regression analysis was performed to find risk factors associated with carotid atherosclerosis. Adjusted variables in multivariable analysis were selected based on a *P* value <0.1 in results from univariable analyses [22]. For longitudinal assessment, subjects who were followed up for more than 5 years were analyzed for the cumulative incidence of carotid plaques. In other words, the carotid plaque is regarded to last after first occurrence during the follow-up. Cumulative incidence of new carotid plaques was analyzed using GEE (Generalized Estimating Equations) in univariable and multivariable analysis. A *P* <0.05 was considered significant. Statistical analysis was performed using statistical analysis system (SAS) software version 9.4 (SAS institute, Cary, NC, USA).

## Results

### Baseline characteristics between subjects with thyroid hormonal status

Among 21,342 subjects (16.3% female) with sustained thyroid functional status for more than 6 months who were included in this study, 365 had subclinical hypothyroidism, 20,927 had euthyroidism, and 50 had subclinical hyperthyroidism (Table 1). The mean age of these groups was 57.4 ± 8.5, 54.1 ± 8.5, and 57.4 ± 7.5 years, respectively. Compared with the euthyroidism group, patients in both the subclinical hypothyroidism and hyperthyroidism group were older. In addition, the subclinical hypothyroidism and subclinical hyperthyroidism groups contained a higher proportion of females than the euthyroidism group. BMI was lower in subjects with subclinical hypothyroidism. In lipid profiles, only TC and LDL-C were significantly different among the three thyroid functional groups. The median follow-up duration was 4.0 years. Baseline characteristics of each group are shown in Table 1.

**Table 1:** Baseline characteristics of subjects with respect to thyroid functional status.

Characteristics	Subclinical hypothyroidism	Euthyroidism	Subclinical hyperthyroidism	*P*
	(*n* = 365)	(*n* = 20,927)	(*n* = 50)	
**Age (years)**	57.4 ± 8.5^a^	54.1 ± 8.5	57.4 ± 7.5^b^	**<0.001**
**Female, n (%)**	87 (23.8)^a^	3,367 (16.1)	23 (46.0)^b^	**<0.001**
**Thyroid status**				
**TSH (mU/L)**	7.98 (6.95, 10.06) ^a^	2.07 (1.41, 2.95)	0.06 (0.02, 0.13) ^b^	**<0.001**
**Free T4 (ng/dL)**	1.15 ± 0.17^a^	1.26 ± 0.18	1.54 ± 0.22^b^	**<0.001**
**Total T3 (ng/dL)**	113 ± 18	114 ± 19	118 ± 21	0.080
**Blood pressure**				
**Systolic (mmHg)**	120.6 ± 15.8	120.7 ± 15.4	119.5 ± 14.6	0.918
**Diastolic (mmHg)**	75.0 ± 11.0	76.0 ± 10.6	72.6 ± 8.9^b^	**0.011**
**BMI (kg/m**^**2**^**)**	23.9 ± 2.5^a^	24.5 ± 2.7	24.4 ± 2.7	**<0.001**
**Microsomal Ab (U/mL)**	6 (1, 99) ^a^	1 [1–14]	1 (1, 25)	**<0.001**
**Lipid profile**				
**Cholesterol (mg/dL)**	193.8 ± 34.6	196.9 ± 34.0	184.1 ± 30.0^b^	**0.006**
**TG (mg/dL)**	133.6 ± 78.5	138.2 ± 81.1	128.8 ± 68.8	0.218
**LDL-C (mg/dL)**	122.2 ± 30.0	125.0 ± 29.8	116.2 ± 26.9	**0.013**
**HDL-C (mg/dL)**	52.8 ± 13.8	52 ± 13.3	49.9 ± 13.0	0.244
**Liver function test**				
**ALT (U/L)**	26.7 ± 43.9^a^	27.2 ± 17.6	28.5 ± 20.1	**0.001**
**AST (U/L)**	26.3 ± 18.3	25.5 ± 11.9	24.9 ± 10.4	0.593
**Glucose**				
**FBS (mg/dL)**	97.0 ± 16.8	98.7 ± 19.9	96.2 ± 10.2	0.289
**HbA1c (%)**	5.69 ± 0.73	5.74 ± 0.83	5.70 ± 0.71	0.823
**Creatinine (mg/dL)**	0.95 (0.83, 1.05)	0.95 (0.85, 1.04)	0.83 (0.71, 0.99) ^b^	**<0.001**
**CRP (mg/dL)**	0.05 (0.03, 0.10)	0.06 (0.03, 0.12)	0.06 (0.03, 0.10)	0.072
**Current smoker, n (%)**	45 (13.2)^a^	5,127 (26.5)	7 (16.3)	**<0.001**
**Exercise (>3/week), n (%)**	101 (28.4)	5,161 (25.8)	11 (23.4)	0.497
**Metabolic syndrome, n (%)**	84 (23.0)	5,679 (27.1)	11 (22.0)	0.154
**Carotid plaques, n (%)**	203 (55.6)^a^	9,993 (47.8)	24 (48.0)	**0.012**

^a^ *P* <0.05 comparison between subclinical hypothyroidism and euthyroidism in post-hoc analysis.

^b^ *P* <0.05 comparison between subclinical hyperthyroidism and euthyroidism in post-hoc analysis.

Continuous data were given as the mean ± SD or median (1^st^,3^rd^ quartile). Nominal data were given as absolute numbers (percentage values). χ^2^-test (nominal data) or Kruskal-Wallis test (continuous data) was performed. Abbreviations: TSH, serum thyrotropin; BMI, body mass index; TG, triglyceride; LDL-C, low-density lipoprotein cholesterol; HDL, high-density lipoprotein cholesterol; AST, aspartate aminotransferase; ALT, alanine aminotransferase; FBS, fasting blood sugar; HbA1c, hemoglobin A1c; CRP, C-reactive protein.

### Factors associated with baseline carotid plaques

The prevalence of carotid plaques at baseline was significantly different among the three groups (55.6% for subclinical hypothyroidism, 47.8% for euthyroidism, 48.0% for subclinical hyperthyroidism; *P* = 0.012). In post-hoc analysis, a difference in prevalence of carotid plaques was found between the subclinical hypothyroidism and euthyroidism groups (*P* = 0.006).

However, when other risk factors were adjusted, FT4 was not a significant risk factor for carotid plaques at baseline in subjects with subclinical hypothyroidism (0.380 [0.097, 1.486]; *P* = 0.164) or euthyroidism (1.094 [0.919, 1.303]; 0.311). Older age, high SBP, high level of serum FBS, and high serum creatinine were independent risk factors for carotid plaques after adjusting of other risk factors in the subclinical hypothyroidism group. Older age, female sex, high SBP, low DBP, high BMI, high level of serum LDL-C, AST, or HbA1c, low level of serum HDL-C, current smoker, and presence of metabolic syndrome were significant risk factors for carotid plaques after adjusting of other risk factors in the euthyroidism group (Table 2).

**Table 2:** Multivariable logistic analysis of risk factor for baseline carotid plaque in each group.

	Euthyroidism^a^		Subclinical hypothyroidism^b^	
	OR (95% CI)	*P*	OR (95% CI)	*P*
**Age (years)**	1.083 (1.079, 1.088)	**<0.001**	1.072 (1.041, 1.104)	**<0.001**
**Female**	1.946 (1.738, 2.180)	**<0.001**	0.823 (0.390, 1.737)	0.61
**Free T4 (ng/dL)**	1.094 (0.919, 1.303)	0.311	0.380 (0.097, 1.486)	0.164
**SBP (mm Hg)**	1.015 (1.011, 1.018)	**<0.001**	1.017 (1.002, 1.032)	**0.023**
**DBP (mm Hg)**	0.992 (0.987, 0.996)	**<0.001**		
**BMI (kg/m**^**2**^**)**	1.020 (1.006, 1.033)	**0.005**		
**TG (mg/dL)**	1.000 (0.999, 1.000)	0.690		
**LDL-C (mg/dL)**	1.005 (1.004, 1.006)	**<0.001**		
**HDL-C (mg/dL)**	0.994 (0.991, 0.997)	**<0.001**		
**ALT (U/L)**	0.998 (0.995, 1.001)	0.283		
**AST (U/L)**	1.005 (1.001, 1.009)	**0.020**		
**FBS (mg/dL)**	1.002 (0.999, 1.004)	0.199	1.022 (1.002, 1.042)	**0.03**
**HbA1c (%)**	1.207 (1.143, 1.274)	**<0.001**	0.719 (0.470, 1.101)	0.129
**Creatinine (mg/dL)**	0.869 (0.696, 1.085)	0.216	13.105 (1.782, 96.359)	**0.012**
**CRP (mg/dL)**	1.075 (0.976, 1.185)	0.141		
**Current smoker**	1.197 (1.111, 1.290)	**<0.001**		
**Exercise (>3/week)**	1.051 (0.979, 1.128)	0.169		
**Metabolic syndrome**	1.130 (1.034, 1.236)	**0.007**		

Multivariable logistic regression analysis was performed in each group. Adjusted variables in multivariable analysis were selected based on a *P* value < 0.1 in results from univariable analyses.

^a^ Multivariable analysis was performed using age, female, free T4, SBP, DBP, BMI, TG, LDL-C, HDL-C, ALT, AST, FBS, HbA1c, creatinine, CRP, current smoker, exercise and metabolic syndrome which was *P* < 0.1 in univariable analysis in euthyroidism subjects.

^b^ Multivariable analysis was performed using age, female, free T4, SBP, FBS, HbA1c and creatinine which was *P* < 0.1 in univariable analysis in subclinical hypothyroidism subjects.

Abbreviations: OR, odd ratio; CI, confidence intervals; TSH, serum thyrotropin; BMI, body mass index; TG, triglyceride; LDL-C, low-density lipoprotein cholesterol; HDL, high-density lipoprotein cholesterol; AST, aspartate aminotransferase; ALT, alanine aminotransferase; FBS, fasting blood sugar; HbA1c, hemoglobin A1c; CRP, C-reactive protein.

In subgroup analysis, there was no significant difference in the carotid plaques at baseline between subjects with severe subclinical hypothyroidism (TSH >10 mU/L, n = 92) and those with euthyroidism (47 [51.1%] subjects with severe subclinical hypothyroidism and 9,993 [47.8%] with euthyroidism; *P =* 0.532 in χ^2^-test).

### Longitudinal assessment of carotid plaques: 5-year follow-up data

The mean follow-up period of the 3,391 subjects included in the longitudinal study was 6.0 years (range, 5 to 7.9 years). They visited the clinic a median of 3 times (range, 2 to 8 times) during the follow-up period at a mean interval of 2.4 years (range, 0.4 to 7.7 years) between each visit.

**Fig 2:**
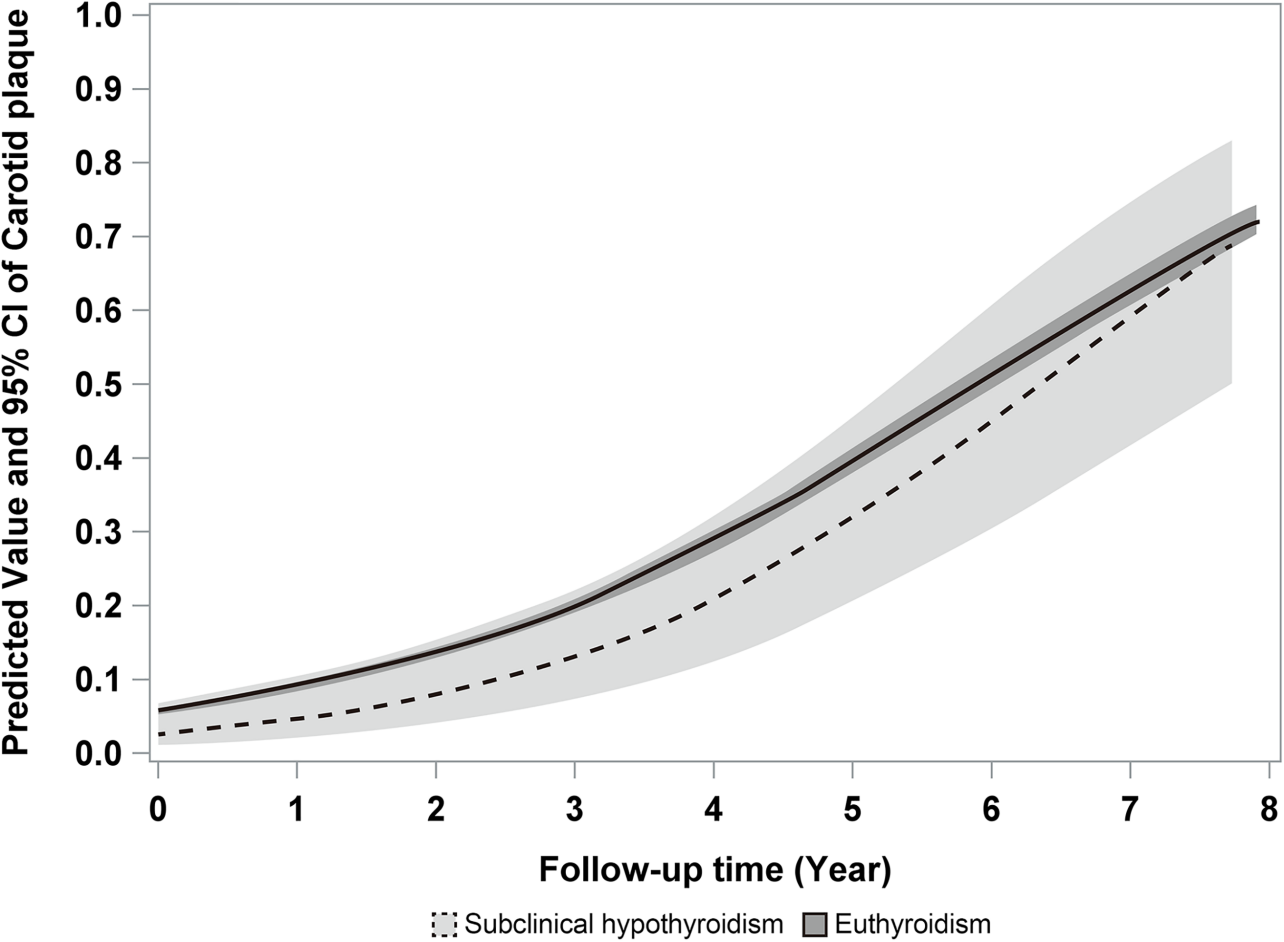
Longitudinal analysis of the cumulative incidence of new carotid plaques during follow-up of more than 5 years. The cumulative incidence of new carotid plaques did not show a significant difference between subclinical hypothyroidism and euthyroidism groups by a generalized estimating equation. CI; confidence interval.

In univariable analysis, follow-up time was significantly associated with cumulative incidence of new carotid plaques (*P* <0.001). However, the cumulative incidence of carotid plaques according to follow-up time was not significantly different between subclinical hypothyroidism and euthyroidism groups (*P* = 0.281; Fig 2). Covariates for multivariable GEE were chosen based on a *P* value <0.1 in univariable analysis (S1 Table). We performed two multivariable GEEs according to the way how to handle thyroid functional status. Effects of TSH and FT4 on cumulative incidence of new carotid plaques were not significantly different with follow-up time (P = 0.518 and P = 0.135, respectively) adjusting for microsomal antibody and smoking status. Effect of thyroid functional status (sustained subclinical hypothyroidism and euthyroidism) on cumulative incidence of new carotid plaques was not significantly different with follow-up time (P = 0.392) adjusting for microsomal antibody and smoking status. Follow-up time was the only factor associated with the cumulative incidence of new carotid plaques after adjustment for other variables (*P* <0.001; Table 3).

**Table 3:** Cumulative incidence of new carotid plaques according to thyroid functional status and follow-up time.

Parameters	TFT as continuous variables			TFT as categorical variables		
	Estimate	95% CI	*p*^a^	Estimate	95% CI	*p*^*b*^
**TSH (mU/L)**	0.0107	-0.0555, 0.0770	0.750			
**Free T4 (ng/dL)**	0.1070	-0.4396, 0.6537	0.701			
**Thyroid functional status**^**c**^				-0.7824	-1.7516, 0.1869	0.114
**Microsomal Ab (U/mL) (/1000)**	-0.0125	-0.0769, 0.0519	0.704	-0.0072	-0.0707, 0.0563	0.825
**Current smoking**	-0.0279	-0.1870, 0.1311	0.731	-0.0300	-0.1886, 0.1287	0.711
**Follow-up time**	0.6089	0.4574, 0.7603	**<0.001**	0.4882	0.4720, 0.5044	**<0.001**
TSH * Follow-up time^**d**^	-0.0041	-0.0167, 0.0084	0.518			
Free T4 * Follow-up time^**d**^	-0.0854	-0.1973, 0.0265	0.135			
**Thyroid functional status**^**c**^ * Follow-up time^**d**^				0.0722	-0.0931, 0.2375	0.392

GEE analysis was performed for multivariable analysis. Adjusted variables in multivariable analysis were selected based on a *P* value <0.1 in results from univariable analyses.

^a^ Analyzed time-dependent differences in the cumulative incidence of carotid plaque according to repeated TSH and free T4 measurements.

^*b*^ Analyzed time-dependent differences in the cumulative incidence of carotid plaque between sustained subclinical hypothyroidism and euthyroidism.

^c^ Subclinical hypothyroidism, compared with euthyroidism.

^d^ Interaction effect with follow-up time.

Abbreviations: CI, confidence interval; TSH, serum thyrotropin.

## Discussion

Carotid atherosclerosis is considered to be an early marker of generalized atherosclerosis [11]. Although overt hypothyroidism is associated with increased carotid atherosclerosis, the effect of subclinical hypothyroidism on carotid atherosclerosis remains controversial. Several studies showed that subclinical hypothyroidism and elevated serum TSH level were associated with increased risk of carotid atherosclerosis, especially in areas of iodine deficiency, and with increased cardiovascular risk [7,12,23,24]. Moreover, in euthyroid subjects, carotid intima-media thickness was inversely associated with low serum FT4 level [25,26]. Cases of severe subclinical hypothyroidism (TSH >10 mU/L) showed clear exacerbation of carotid atherosclerosis [7,27]. Monzani *et al*. reported that the treatment of patients with subclinical hypothyroidism with levothyroxine significantly improved carotid atherosclerosis [28]. In contrast, some studies showed that subclinical hypothyroidism was not related to increased risk of carotid atherosclerosis, but rather to decreased carotid artery intima-media thickness [6,29,30]. Other studies did not show a significant association between subclinical thyroid disease and cardiovascular risk [31–33]. There also has been debate about the increased risk of carotid atherosclerosis in cases of subclinical hyperthyroidism [6,29,30].

We thought that certain factors may interfere with the effect of thyroid dysfunction on carotid atherosclerosis. Notably, subclinical thyroid dysfunction can improve or worsen during the follow-up period [13–17,34]. Therefore, the use of a single set of TFTs could result in inclusion of transient subclinical thyroid dysfunction, and this may weaken the effects of subclinical thyroid dysfunction on carotid atherosclerosis. Furthermore, this classification may lead to inaccurate longitudinal assessment. When excluding transient subclinical thyroid dysfunction, the effects of sustained subclinical thyroid dysfunction on carotid atherosclerosis can be assessed more accurately. Previously, two studies investigated the effects of persistent subclinical hypothyroidism on cardiovascular risk [35,36]. In a study of elderly subjects, persistent subclinical hypothyroidism was not associated with the incidence of coronary heart disease, heart failure, or cardiovascular death. However, in another study conducted in children, clinical and biochemical cardiovascular risk factors were significantly associated with long standing subclinical hypothyroidism.

In our study, to reduce such confounders, we selected subjects with sustained thyroid hormone status for more than 6 months. We excluded 1,587 subjects with transient thyroid dysfunction. The effect of thyroid function was adjusted for other risk factors for carotid plaques. As a result, sustained subclinical thyroid dysfunction was not an independent risk factor for the presence of carotid plaques at baseline. To confirm the result of cross-sectional analysis for pre-existing carotid plaques, we performed longitudinal analysis in subjects that were followed up for more than 5 years and calculated the cumulative incidence of new plaques. A generalized estimating equation was used to evaluate the interaction between the thyroid functional status and follow-up time. TSH/FT4 values and sustained subclinical hypothyroidism did not significantly affect the cumulative incidence of new carotid plaques according to follow-up time. The results remained unchanged in subgroup analysis of severe subclinical hypothyroidism and euthyroidism.

There are several limitations to this study. First, subjects of our study were enrolled from a health screening center in a tertiary hospital and subjects were self-selected for the examination. Therefore, selection bias and the “healthy worker effect” could have affected our results. People with carotid atherosclerosis and those at high risk for CVD tend to have repeated carotid DUS. However, large longitudinal cohort studies of subclinical thyroid dysfunction and carotid atherosclerosis are difficult to perform due to the limitation of case enrollment. Therefore, we selected subjects who underwent serial TFT and carotid DUS for more than 5 years during health check-ups. Second, there were relatively few subjects with sustained subclinical hypothyroidism and hyperthyroidism compared to the euthyroidism group. Since we hypothesized that the effects of sustained subclinical thyroid dysfunction on carotid atherosclerosis would be more consistent and clear than those of transient subclinical thyroid dysfunction, we only included cases with sustained thyroid function and thus the incidence of subclinical hypothyroidism in our study was lower than seen in the general population. Third, although drug and disease status could affect the occurrence of carotid plaques, but they are not included in the analysis. Instead, we included blood pressure, lipid profile, liver function test, glucose, creatinine and metabolic syndrome as variables. Lastly, this was a retrospective study and prospective cohort studies with serial measurement of TFT and carotid DUS could provide more information on this topic.

In summary, sustained subclinical thyroid dysfunction was not an independent risk factor for carotid plaques at baseline in healthy individuals. Furthermore, in longitudinal assessment, subclinical thyroid dysfunction was not associated with the cumulative incidence of new carotid plaques, and only longer follow-up time was a significant predictor of incident carotid plaques.

## Supporting information

S1 Table Difference in baseline characteristics between subclinical hypothyroidism and euthyroidism groups among subjects who were followed up for more than 5 years. (DOCX)
