# Supplementary material for: Subclinical thyroid dysfunction and risk of carotid atherosclerosis
Source: PLoS One. 2017 Jul 27;12(7):e0182090. doi: 10.1371/journal.pone.0182090 (PMC5531563; doi:10.1371/journal.pone.0182090)
Supplement: S1 Table — (DOCX) [file pone.0182090.s001.docx]

**S1 Table. Difference in baseline characteristics between subclinical hypothyroidism and euthyroidism groups among subjects who were followed up for more than 5 years.**

| **Characteristics** | **Subclinical hypothyroidism** | **Euthyroidism** | ***P*** |
| --- | --- | --- | --- |
|  | **(*n* = 45)** | **(*n* = 3,346)** |  |
| **Age (years)** | 54.1 ± 8.0 | 53.2 ± 7.9 | 0.465 |
| **Female, n (%)** | 6 (13.3) | 488 (14.6) | 0.813 |
| **Thyroid status** |  | | |
| **TSH (mU/L)** | 8.43 (7.46, 10.6) | 2.05 (1.44, 2.9) | **<0.001** |
| **Free T4 (ng/dL)** | 1.17 ± 0.16 | 1.27 ± 0.18 | **<0.001** |
| **Total T3 (ng/dL)** | 111 ± 13 | 114 ± 20 | 0.429 |
| **Blood pressure** |  | | |
| **Systolic (mmHg)** | 120.6 ± 15.0 | 118.0 ± 14.5 | 0.233 |
| **Diastolic (mmHg)** | 76.2 ± 9.2 | 74.4 ± 10.3 | 0.193 |
| **BMI (kg/m^2^)** | 24.6 ± 2.6 | 24.5 ± 2.6 | 0.612 |
| **Microsomal Ab (U/mL)** | 1(1, 461) | 1 (1, 1) | **<0.001** |
| **Lipid profile** |  | | |
| **Cholesterol (mg/dL)** | 195.1 ± 28.3 | 194.8 ± 32.5 | 0.923 |
| **TG (mg/dL)** | 140.2 ± 81.9 | 139.3 ± 81.2 | 0.987 |
| **LDL-C (mg/dL)** | 125.4 ± 25.2 | 124.2 ± 28.1 | 0.695 |
| **HDL-C (mg/dL)** | 51.8 ± 13.9 | 52.7 ± 13.4 | 0.462 |
| **Liver function test** |  | | |
| **ALT (U/L)** | 23.3 ± 10.2 | 26.7 ± 17.1 | 0.270 |
| **AST (U/L)** | 22.6 ± 5.2 | 24.9 ± 9.9 | 0.192 |
| **Glucose** |  | | |
| **FBS (mg/dL)** | 92.8 ± 10.3 | 96.0 ± 16.7 | 0.315 |
| **HbA1c (%)** | 5.51 ± 0.63 | 5.55 ± 0.72 | 0.787 |
| **Creatinine (mg/dL)** | 0.97 (0.91, 1.07) | 0.97(0.87, 1.05) | 0.409 |
| **CRP (mg/dL)** | 0.06 (0.03, 0.10) | 0.06 (0.03, 0.11) | 0.757 |
| **Current smoker, n (%)** | 5 (12.8) | 783 (25.2) | 0.077 |
| **Exercise (>3/week), n (%)** | 8 (17.8) | 870 (27.0) | 0.165 |
| **Metabolic syndrome, n (%)** | 9 (20.0) | 774 (23.1) | 0.620 |

Continuous data were given as the mean ± SD or median (IQR). Nominal data were given as absolute numbers (percentage values). χ^2^-test (nominal data) or Wilcoxon rank sum test (continuous data) were performed. TSH, serum thyrotropin; BMI, body mass index; TG, triglyceride; LDL-C, low-density lipoprotein cholesterol; HDL, high-density lipoprotein cholesterol; AST, aspartate aminotransferase; ALT, alanine aminotransferase; FBS, fasting blood sugar; HbA1c, hemoglobin A1c; CRP, C-reactive protein.
